# Supplementary material for: Covalent targeting as a common mechanism for inhibiting NLRP3 inflammasome assembly
Source: bioRxiv. 2023 Jun 1:2023.06.01.543248. Preprint. [Version 1] doi: 10.1101/2023.06.01.543248 (PMC10312593; doi:10.1101/2023.06.01.543248)
Supplement: Supplement 1 [file NIHPP2023.06.01.543248v1-supplement-1.pdf]

bioRxiv preprint doi: https://doi.org/10.1101/2023.06.01.543248; this version posted June 1, 2023. The copyright holder for this preprint (which was not certified by peer review) is the author/funder, who has granted bioRxiv a license to display the preprint in perpetuity. It is made available under aCC-BY 4.0 International license.

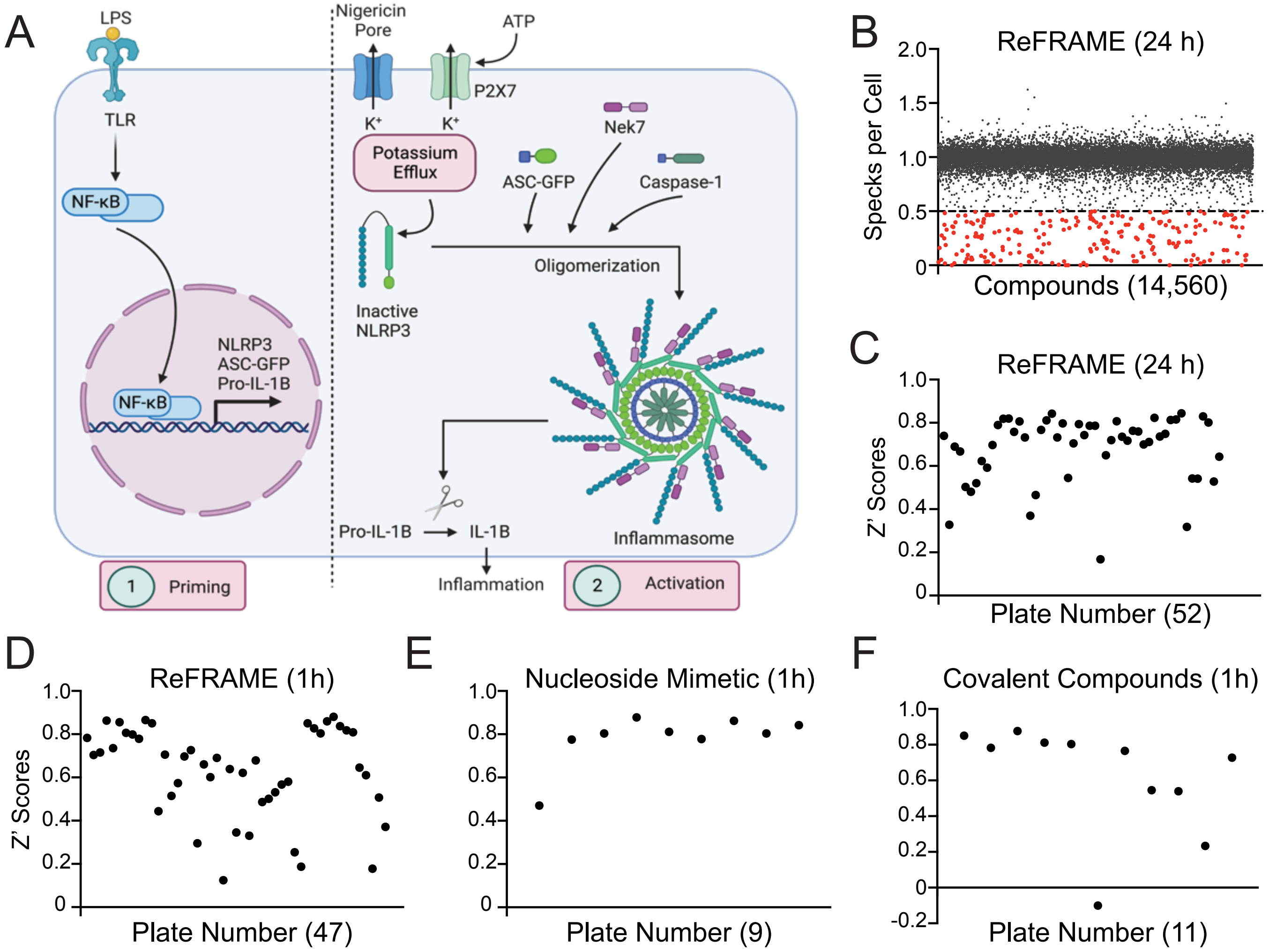

Supplementary Figure 1: A High Throughput Screen for Inhibitors of ASC-Speck Formation

(A) Schematic of NLRP3 inflammasome activation including ASC-GFP. (B) Scatterplots of specks per cell evaluated via high throughput screen for inhibitors of inflammasome assembly for compounds tested in 24 hr ReFRAME Screen. (C-F) Z' Values for plates from 24 hr ReFrame Screen (C), 1 hr ReFrame Screen (D), 1 hr Nucleoside Mimetic Screen (E), and 1 hr Covalent Library Screen (F).

bioRxiv preprint doi: https://doi.org/10.1101/2023.06.01.543248; this version posted June 1, 2023. The copyright holder for this preprint (which was not certified by peer review) is the author/funder, who has granted bioRxiv a license to display the preprint in perpetuity. It is made available under aCC-BY 4.0 International license.

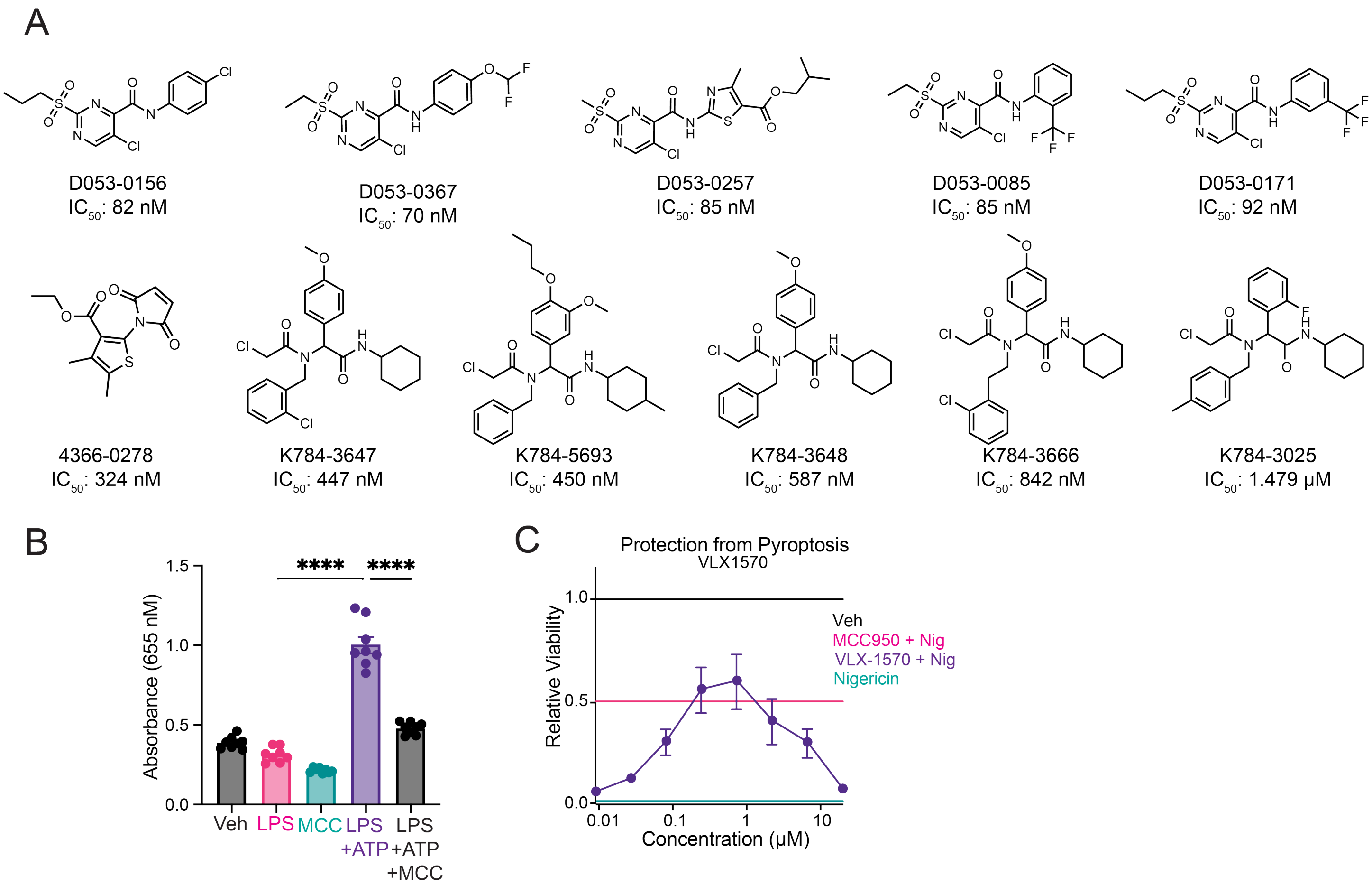

Supplementary Figure 2: Identified hit compound classes are active in secondary assays of NLRP3 inflammasome activity.

(A) Structures representing hit compounds profiled from the 9 covalent scaffolds identified. (B) IL-1β secretion inhibition in WT THP-1 cells stimulated with LPS and ATP and measured by SEAP secretion from HEK-Blue-IL-1β reporter cells. Error bars show SEM for n = 8 replicates. ****$p<0.0001$ for ordinary one-way analysis of variance (ANOVA) with Tukey correction for multiple comparisons between conditions. (C) Representative protection from NLRP3-mediated pyroptotic cell death induced by LPS and Nigericin in WT THP-1 by VLX1570 in dose response. Error bars show SEM for n = 4 replicates.

bioRxiv preprint doi: https://doi.org/10.1101/2023.06.01.543248; this version posted June 1, 2023. The copyright holder for this preprint (which was not certified by peer review) is the author/funder, who has granted bioRxiv a license to display the preprint in perpetuity. It is made available under aCC-BY 4.0 International license.

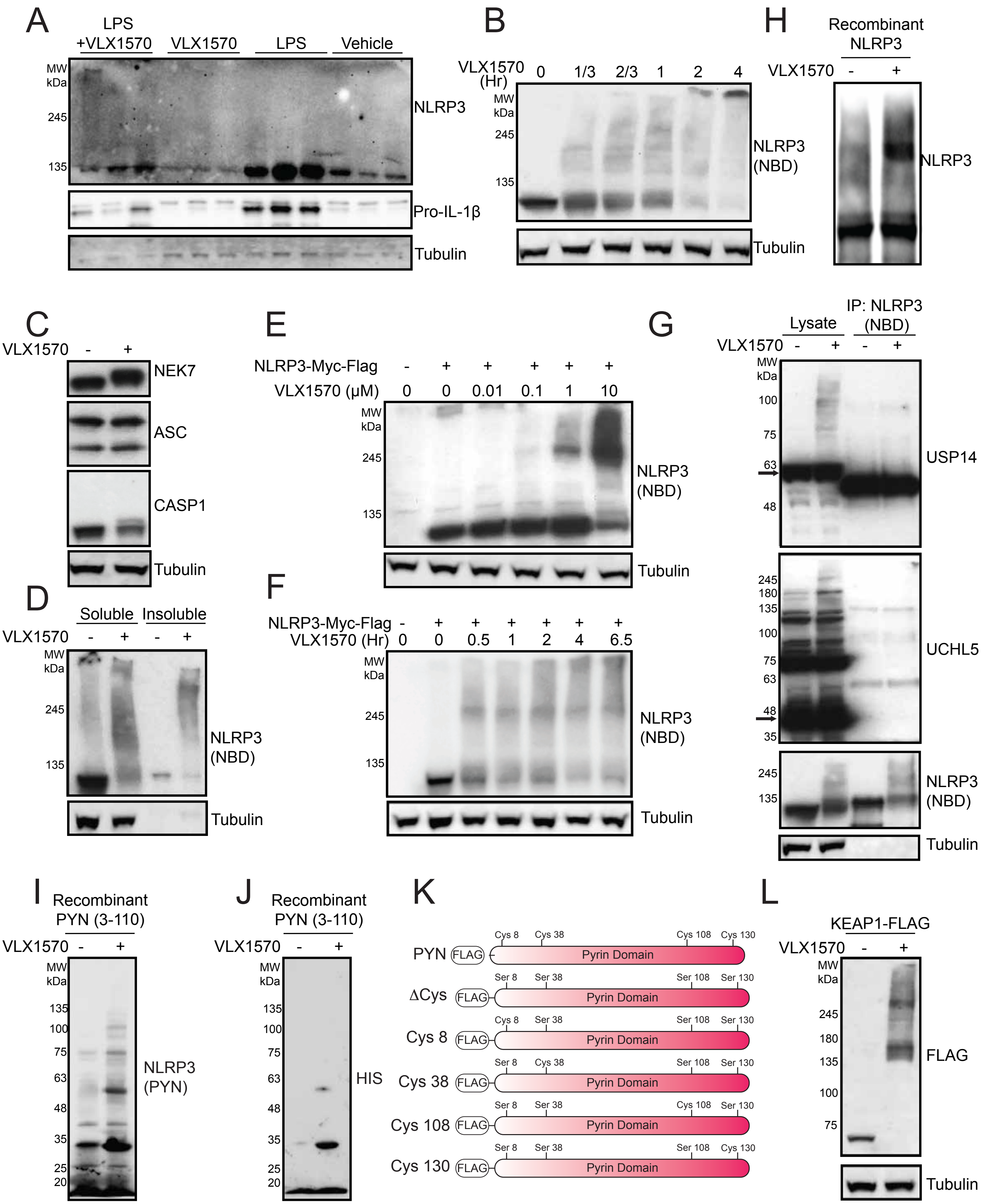

bioRxiv preprint doi: https://doi.org/10.1101/2023.06.01.543248; this version posted June 1, 2023. The copyright holder for this preprint (which was not certified by peer review) is the author/funder, who has granted bioRxiv a license to display the preprint in perpetuity. It is made available under aCC-BY 4.0 International license.

Supplementary Figure 3. VLX1570 covalently modifies and crosslinks NLRP3.

(A) Western blot of NLRP3, Pro-IL-1β, and Tubulin in cells harvested from mouse peritoneal lavage fluid of mice stimulated with 10 mg/kg LPS (3 hr), pretreated with 4.4 mg/kg VLX1570 1 hr prior to LPS injection. (B) Western blot of NLRP3 (NBD) and Tubulin from THP-1 cells treated with 10 μM VLX1570 at indicated time points. (C) Western blot for NEK7, ASC, CASP1 and Tubulin in THP-1 cells treated with 10 μM VLX1570 for 2 hr. (D) Western blot for NLRP3 (NBD) and Tubulin in soluble and insoluble fractions from lysates of WT THP-1 cells treated with 10 μM VLX1570 for 2 hr. (E) Western blot of NLRP3 (NBD) from HEK293T cells overexpressing NLRP3-Myc-FLAG treated in dose response with VLX1570 for 2 hr. (F) Western blot of NLRP3 (NBD) from HEK293T cells overexpressing NLRP3-Myc-FLAG treated in with 10 μM VLX1570 at indicated time points. (G) Western blot of USP14, UCHL5, NLRP3 (NBD), and Tubulin from WT THP-1 lysate or NLRP3-immunoprecipated material from WT THP-1 cells with or without VLX1570 treatment. (H) Western blot of NLRP3 (NBD) from recombinant WT NLRP3 treated with 50 μM VLX1570 at 4 °C overnight. (I-J) Western blot of NLRP3 (PYN) and HIS-TAG from recombinant NLRP3 PYN (3-110, C08S, C38S) treated with 50 μM VLX1570 at 4 °C overnight. (K) NLRP3 PYN Domain ΔCYS or Single CYS constructs. (L) Western blot of FLAG from HEK293T cells overexpressing KEAP1-FLAG treated with or without VLX1570 (10 μM) for 2 hr.

bioRxiv preprint doi: https://doi.org/10.1101/2023.06.01.543248; this version posted June 1, 2023. The copyright holder for this preprint (which was not certified by peer review) is the author/funder, who has granted bioRxiv a license to display the preprint in perpetuity. It is made available under aCC-BY 4.0 International license.

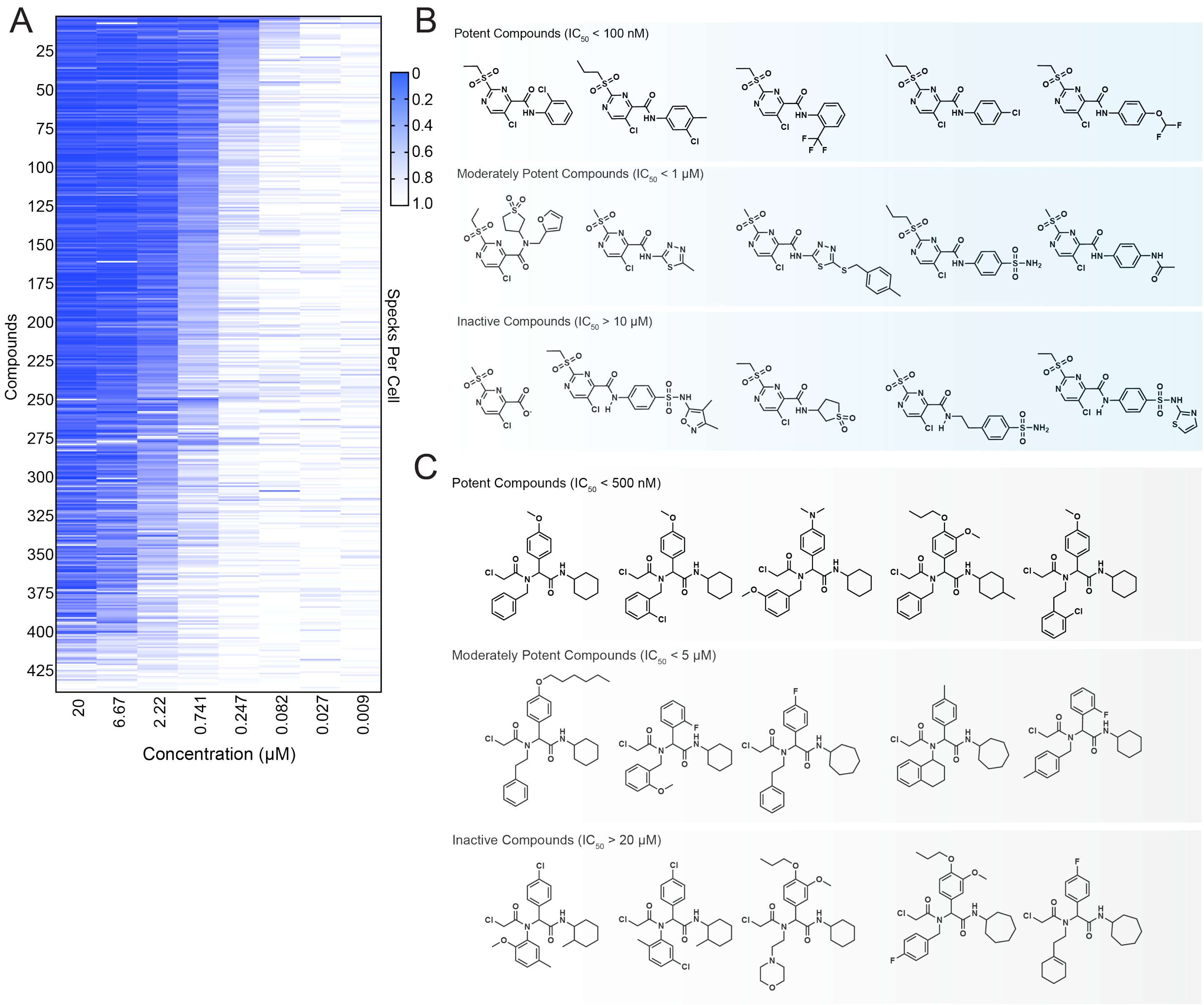

Supplementary Figure 4: Structure activity relationship profiling of 2-sulfonylpyrimidine and chloroacetamide scaffolds.

(A) Structure activity relationship for ASC-Speck Assay for chloroacetamide scaffold by supplier inventory. (B) Structure of 2-sulfonylpyrimidine compounds which are potent (IC_{50} < 100 nM), moderately active (IC_{50} < 1 μM), or inactive (IC_{50} > 10 μM) in the ASC-Speck Assay. (C) Structure of chloroacetamide compounds which are potent (IC_{50} < 500 nM), moderately active (IC_{50} < 5 μM), or inactive (IC_{50} > 20 μM) in the ASC-Speck Assay.

bioRxiv preprint doi: https://doi.org/10.1101/2023.06.01.543248; this version posted June 1, 2023. The copyright holder for this preprint (which was not certified by peer review) is the author/funder, who has granted bioRxiv a license to display the preprint in perpetuity. It is made available under aCC-BY 4.0 International license.

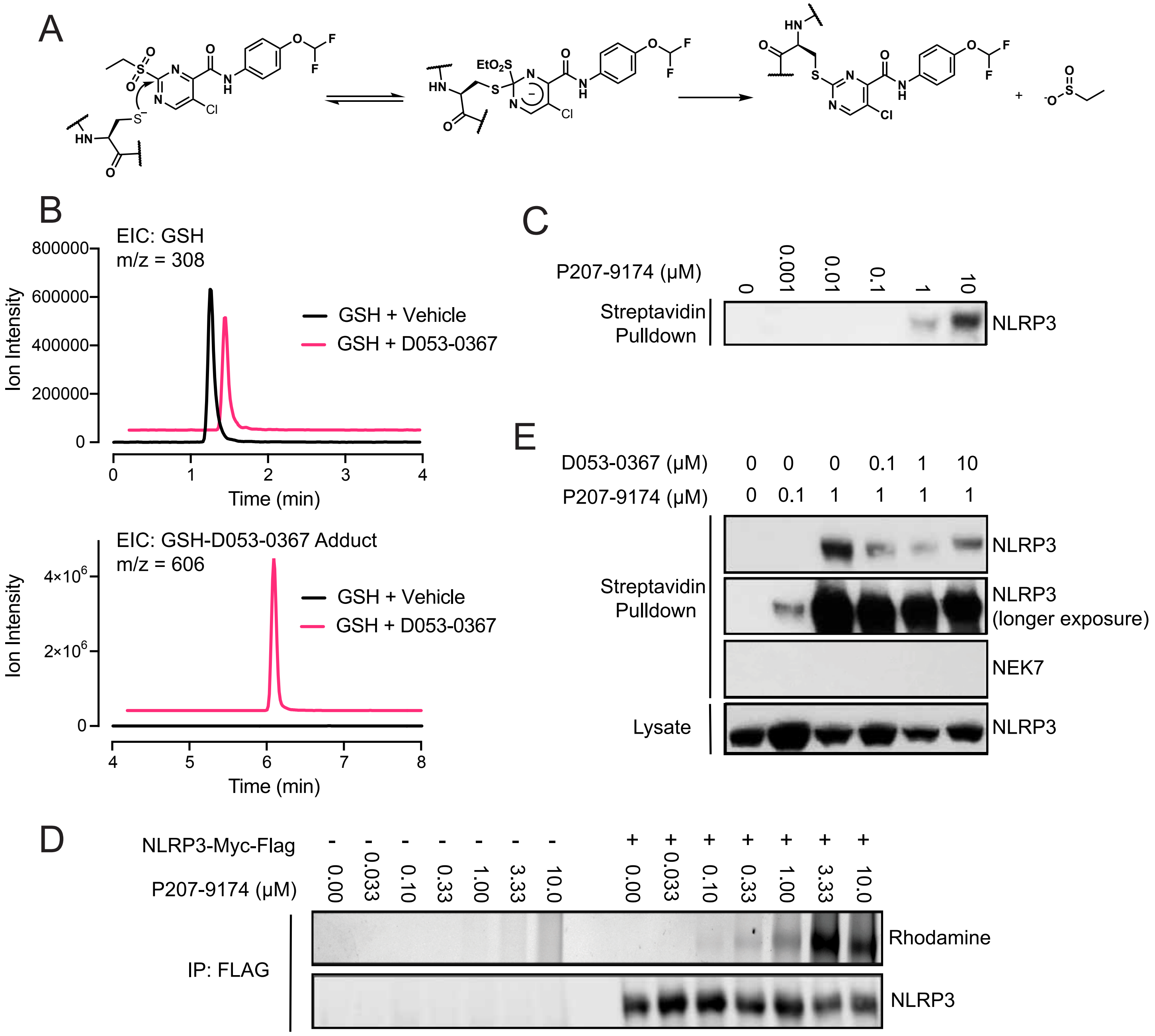

Supplementary Figure 5: A 2-sulfonylpyrimidine-containing inhibitor of NLRP3 inflammasome formation that covalently modifies cysteines via an S_NAr-based mechanism.

(A) S_NAr Covalent mechanism for 2-sulfonylpyrimidine scaffold. (B) LC-MS quantification of GSH (4 mM), D053-0367 (1 mM), or D053-0367 (1 mM) incubated in the presence or absence of GSH (4 mM) in PBS for 1 hour. Relative ion intensities were compared with chromatograms shown. (C) Anti-NLRP3 western blot of streptavidin-enriched material after in situ treatment of THP-1 cells with P207-9174 in dose response for 1 hr. (D) Anti-NLRP3 Western blot and rhodamine imaging of FLAG immunoprecipitated material after in situ treatment of NLRP3-overexpressing HEK293T cells with P207-9174 in dose response for 1 hr. (E) Anti-NLRP3 and Anti-NEK7 Western blot of streptavidin-enriched material after in situ treatment of THP-1 cells with P207-9174 for 1 hr competed with D053-0367 pretreated for 1 hr.

bioRxiv preprint doi: https://doi.org/10.1101/2023.06.01.543248; this version posted June 1, 2023. The copyright holder for this preprint (which was not certified by peer review) is the author/funder, who has granted bioRxiv a license to display the preprint in perpetuity. It is made available under aCC-BY 4.0 International license.

Supplementary Table 1:

**Compound**	**Source**	**Catalog Number**
VLX1570	Cayman Chemical	29721-10
Bay 11-7082	Fisher Scientific	T2846100MG
Oridonin	SelleckChem	S2335
Rrx-001	MedChemExpress	HY-16438
Omaveloxolone	Fisher Scientific	501151750
Erianin	Cayman Chemical	32516-10
MNS	Cayman Chemical	15206-10
4-octyl Itaconate	Cayman Chemical	25374-25
Withaferin A	Cayman Chemical	11352-10
Diroximel Fumarate	Cayman Chemical	29111-5
BCI (hydrochloride)	Cayman Chemical	21945-5

Supplementary Table 2:

Protein	Supplier	Catalog Number	Host Species	Dilution
NLRP3 (NBD)	Cell Signaling	15101S	Rabbit	1:1000 (BSA)
NLRP3 (PYN)	Adipogen	AG-20B-0014-C100	Mouse	1:1000 (BSA)
Tubulin	Sigma	T6557	Mouse	1:2000 (BSA)
FLAG	Sigma	F1804	Mouse	1:1000 (Milk)
IL-1β	GeneTex	GTX74034	Rabbit	1:1000 (BSA)
GFP	Abcam	Ab290	Rabbit	1:1000 (BSA)
USP14	Cell Signaling	11931S	Rabbit	1:1000 (BSA)
UCHL5	Santa Cruz	sc-271002	Mouse	1:1000 (BSA)
ASC	Cell Signaling	13833S	Rabbit	1:1000 (BSA)
CASP1	Cell Signaling	3866S	Rabbit	1:1000 (BSA)
NEK7	Cell Signaling	3057S	Rabbit	1:1000 (BSA)
